# Recurrent *FGFR2* and *PIK3CA* Mutations in Sialoblastoma

**DOI:** 10.1007/s12105-025-01838-3

**Published:** 2025-09-04

**Authors:** Selene C. Koo, Jingqun Ma, Quynh T. Tran, Rita Alaggio, Alessandra Stracuzzi, Pauline M. Chou, Benjamin J. Wilkins, Brent A. Orr, Faizan Malik

**Affiliations:** 1 Department of Pathology, St. Jude Children’s Research Hospital, Memphis, TN USA; 2 Pathology and Oncology Unit, Ospedale Pediatrico Bambino Gesù, IRCCS, Rome, Italy; 3 Department of Pathology, Ann and Robert H. Lurie Children’s Hospital of Chicago, Chicago, IL USA; 4 Department of Pathology and Laboratory Medicine, Children’s Hospital of Philadelphia, Philadelphia, PA USA

**Keywords:** Sialoblastoma, Salivary gland, Congenital, *FGFR2*, *PIK3CA*, *CTNNB1*

## Abstract

**Purpose:**

Sialoblastoma is an extremely rare low-grade malignant salivary gland neoplasm that presents at birth or early infancy and has heterogeneous clinical behavior. Due to its rarity, the molecular landscape remains incompletely characterized. We aimed to expand the current understanding of the genetic alterations in sialoblastoma through comprehensive molecular analysis.

**Methods:**

Five sialoblastoma cases were retrieved from four institutional archives. Clinical and pathologic review was performed, and targeted next-generation sequencing was conducted using clinically validated panels. Copy number analysis was performed on four cases.

**Results:**

The cohort included five patients with tumors located in parotid gland (*n* = 2), minor salivary glands (*n* = 2), and submandibular gland (*n* = 1). Four patients were diagnosed before 6 months of age. Histologically, all tumors showed solid organoid nests with primitive basaloid cells, dense fibrous stroma, and mitotic activity ranging from 8 to 25 per 10 high-power fields. Recurrent *FGFR2* p.C382R variants were identified in 80% (4/5) of cases. Additional alterations were seen in *FGFR2* p.C382R mutated tumors, including *PIK3CA* hotspot mutations in two cases (p.R88Q, p.R38H) and a truncating *FGFR2* variant (p.L776Rfs) in one. The single tumor that lacked *FGFR2* mutations harbored a *CTNNB1* p.I35T variant and showed more favorable histologic features. Copy number analysis revealed recurrent whole-chromosome gains of chromosomes 8, 10, and 11.

**Conclusion:**

A distinct subset of sialoblastoma has *FGFR2* p.C382R hotspot mutation as the predominant driver mutation. Tumors with this mutation tend to have solid growth pattern, aggressive histologic features, and clinical behavior. The identification of concurrent genomic alterations expands the molecular landscape of this rare tumor. The detection of alternative drivers, such as *CTNNB1* hotspot mutations typical of basal cell adenoma, also suggests that a subset of sialoblastoma may represent other salivary gland tumors presenting in infancy.

## Introduction

Sialoblastoma is an extremely rare low-grade malignant salivary gland neoplasm that presents at birth or in early infancy and has heterogeneous clinical behavior [1, 2]. Histologically, it resembles primitive salivary gland structures, with solid, organoid arrangement of basaloid cells. Histologic features that correlate with aggressive behavior include necrosis, increased mitotic activity, and high Ki-67 proliferation index [3]. Because of its extreme rarity, the molecular landscape of sialoblastoma has not been fully characterized. Previous studies have identified *FGFR2*, *PIK3CA*, and *HRAS* mutations as potential oncogenic drivers in single cases [4–6]. Jia et al. recently showed that sialoblastomas harboring *FGFR2* mutations (~ 60% of cases) display aggressive histologic features [4].

Given the known aggressive behavior and the potential for intervention with targeted therapeutics, additional molecular profiling to characterize this rare tumor is critical. In this study, we comprehensively analyzed the histologic and molecular features of five additional sialoblastomas to further explore their diversity.

## Materials/Methods

### Case Selection and Histologic Review

After Institutional Review Board approval, all cases previously diagnosed as sialoblastoma were retrieved from the archives of four institutions. Hematoxylin and eosin (H&E) and immunohistochemical stains were performed according to clinically validated protocols at each institution and were subsequently reviewed by two pathologists to confirm the diagnosis. Ki67 proliferative index was manually estimated. Pertinent clinical information, imaging, and follow-up was collected. Only tumors with available material for molecular testing were included in the cohort.

### Targeted Gene Sequencing

Targeted next-generation sequencing (NGS) was performed on formalin-fixed, paraffin-embedded (FFPE) tissue from all tumors using one of three clinically validated sequencing assays: a whole exome-based (Illumina TruSeq DNA Exome) next generation sequencing panel with enhanced target capture of 301 cancer genes (Patients 1, 4, and 5), a targeted gene sequencing panel of 362 pediatric cancer genes using Twist (Twist Bioscience, South San Francisco, CA) target enrichment (Patient 3; [7]), or a commercially available targeted sequencing assay of 523 cancer-relevant genes (TruSight Oncology500 Panel, Illumina; Patient 2).

### Methylation Array Copy Number Analysis

DNA from tumors from Patients 1, 3, 4, and 5 was extracted and hybridized to Illumina Infinium HumanMethylationEPICv2 BeadChip arrays. All methylation array data analysis was performed in R (http://www.r-project.org, version 4.4.0), using several packages from Bioconductor and other repositories. Specifically, array data were preprocessed using the *minfi* package (v.1.28.4) [8]. Background correction with dye-bias normalization was performed for all samples using noob (normal-exponential out-of-band) with “single” dye method [9].

Copy number variation (CNV) analysis from methylation array data was performed using the *conumee* package (version 1.16.0) [10]. Chromosomal gain or loss was determined using a 0.18 threshold.

All molecular results were interpreted by a board-certified molecular pathologist (S.C.K.).

## Results

Clinical and demographic information is summarized in Table 1. Tumors from five patients (3 female, 2 male) were included. Tumors were located in the parotid gland (*n* = 2; Patients 2 and 4), minor salivary glands (*n* = 2; Patient 1, lip commissure; Patient 3, buccal), and submandibular gland (*n* = 1; Patient 5). Tumor size ranged from 1.5 to 7.0 cm. Four patients were diagnosed before 6 months of age, while one was diagnosed at the time of tumor recurrence at 4 years of age (Patient 3; information on primary tumor is unavailable). One patient (Patient 5) concurrently developed a liver tumor, which was diagnosed as hepatoblastoma (epithelial type, fetal pattern, PRETEXT [PRE-Treatment EXTent of tumor] III due to involvement of right anterior and left medial and lateral liver segments [11]). Follow-up was available in 2 patients (20 months to 10 years). Patient 1 developed local recurrence at 5 months of age, was treated with chemotherapy (carboplatin/etoposide/doxorubicin), and had stable disease at last follow-up (20 months after initial diagnosis). Patient 3 was treated with resection only and had no evidence of recurrent disease after 10-year follow-up.

**Fig. 1:**
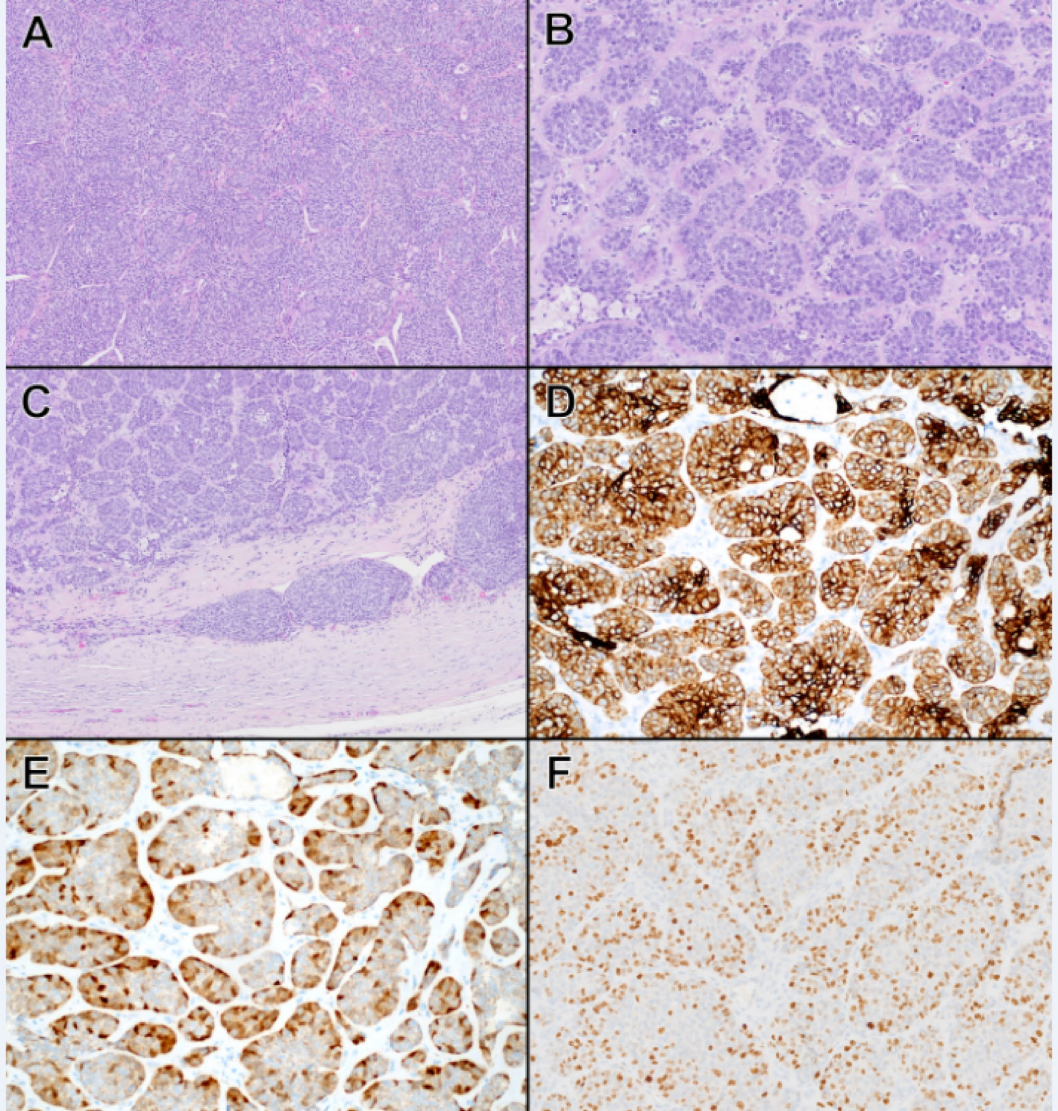
Histologic findings in a sialoblastoma with *FGFR2* p.C382R and *PIK3CA* mutation (Patient 1). **A**. The tumor consists of back-to-back nests of basaloid cells with intervening thin-walled blood vessels (H&E, 100x magnification). Tumor cells show mild nuclear pleomorphism and scant amphophilic cytoplasm, with abundant mitotic figures, focal comedo-like necrosis (**B**), and focal lymphovascular invasion (**C**) (**B**, H&E, 200x magnification; C, H&E, 100x magnification). **D** Tumor cells are diffusely positive for cytokeratin CAM5.2 (**D**) and S100 (**E**), with stronger CAM5.2 positivity near the central/luminal area and more distinct nuclear S100 positivity along the periphery of tumor nests (**D**, CAM5.2, and** E**, S100, 200x magnification). **F** Ki67 proliferative index in the initial tumor is high (approximately 60%) in tumor cells (Ki67, 200x magnification).

**Fig. 2:**
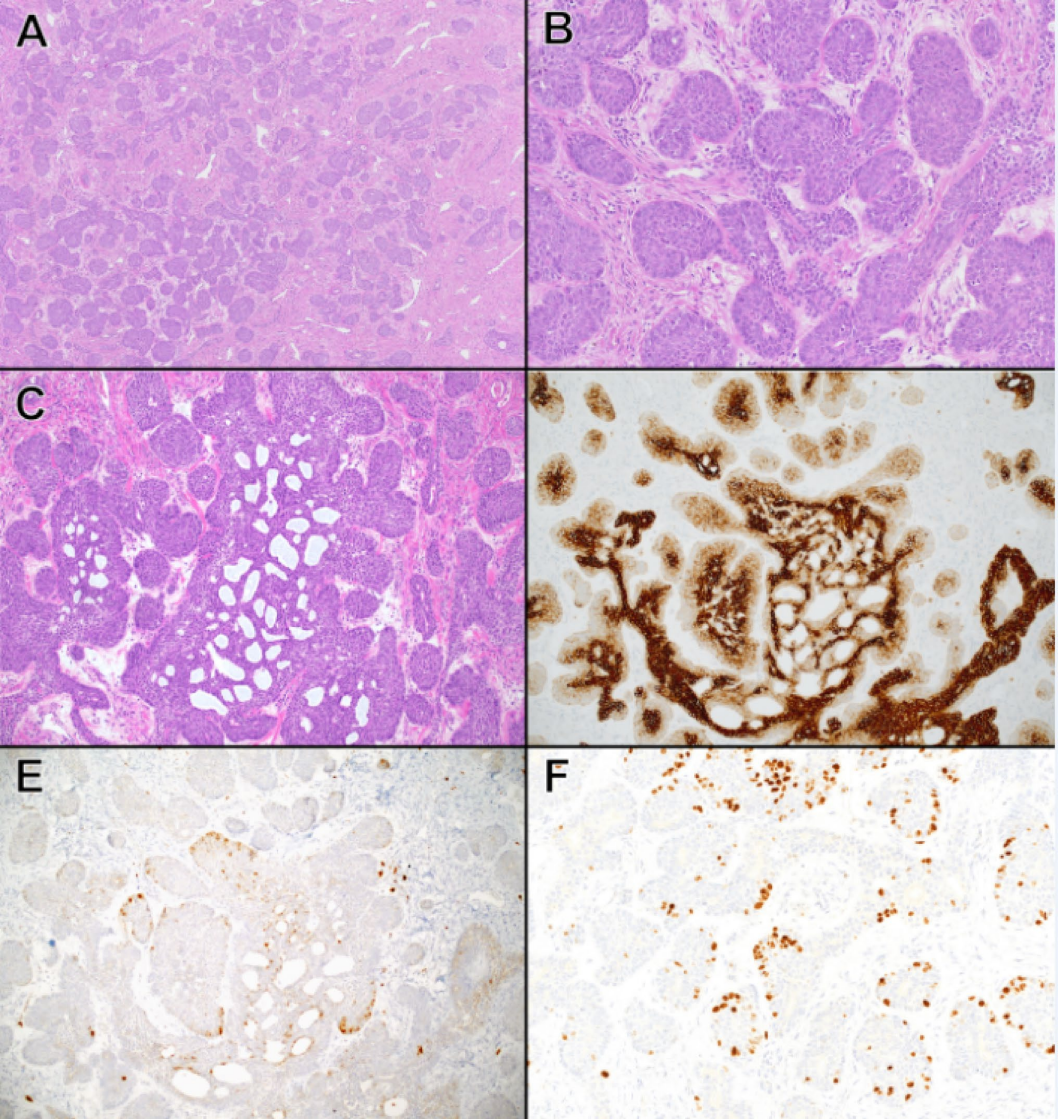
Histologic findings in a sialoblastoma with *CTNNB1* mutation and concurrent hepatoblastoma (Patient 5). **A** The tumor shows abundant collagenized stroma separating nests of basaloid cells and interspersed glandular elements (H&E, 50x magnification). Tumor nests are largely solid, with some nests adopting more glandular-type differentiation (**B**) and areas of cribriform architecture (**B** H&E, 200x magnification; **C** H&E, 100x magnification). **D** Cytokeratin CAM5.2 is diffusely positive in tumor cells, with stronger luminal cell positivity and weaker positivity at the periphery of tumor nests (CAM5.2, 100x magnification). **E** S100 shows focal positivity in tumor cells, largely along the periphery of tumor nests (S100, 100x magnification). **F** Ki67 proliferative index in the tumor is variable, overall approximately 20% in tumor cells (Ki67, 200x magnification)

Histologically, all five tumors showed solid organoid nests of tumor cells separated by dense fibrous stroma and supported by thin-walled branching blood vessels. The tumor cells had a primitive, basaloid appearance and contained scant cytoplasm with mild nuclear irregularity and few prominent nucleoli (Figs. 1 and 2). Mitotic activity ranged from 8 to 25 figures per 10 high-power fields (HPF). Tumor necrosis, typically focal and comedo-like, was seen in three tumors (Patients 1, 2, and 3). The capsule was assessable in 3 cases; no invasion was appreciated. Lymphovascular invasion was seen in two cases (Patients 1 and 2). Immunohistochemically, the tumor cells were positive for cytokeratins (5/5, diffuse positivity with stronger expression in the center of tumor nests), epithelial membrane antigen (1/1), and S100 (5/5, with accentuation in the periphery of tumor nests) (Figs. 1D–E and 2D–E). Smooth muscle actin was negative in all tested tumors (0/3). P63 stain was performed in three cases (Patients 1, 3, and 4), with near-diffuse positivity in all three cases. Beta-catenin was performed in 1 case (Patient 1) and did not show nuclear localization. Ki-67 nuclear labeling ranged from 20 to 60% (Fig. 1F, 2 F).

## Molecular Findings

**Fig. 3:**
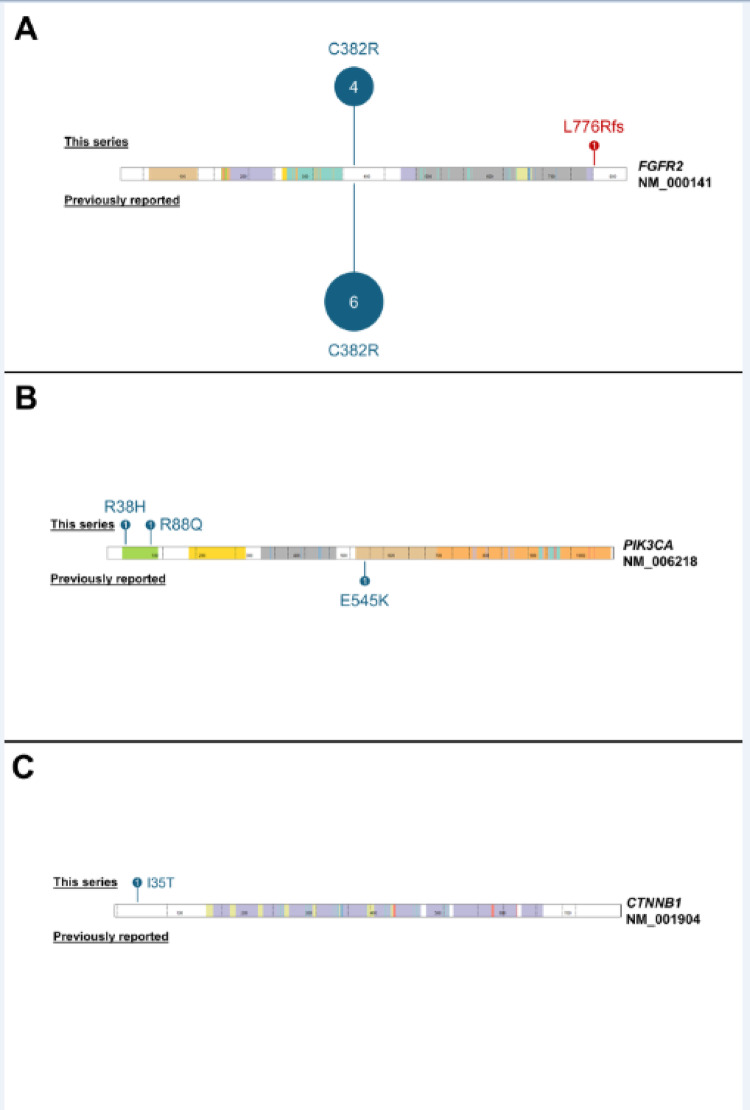
Lollipop plot demonstrating spectrum of mutations in *FGFR2* (**A**), *PIK3CA* (**B**), and *CTNNB1* (**C**) in sialoblastomas.

Sequencing results are summarized in Table 1; Fig. 3. Identical *FGFR2* p.C382R variants were detected in four of five cases (Patients 1–4). In addition to the *FGFR2* p.C382R variant, three cases (Patients 1–3) had additional sequence variants, including *PIK3CA* hotspot variants in two cases (Patient 1: *PIK3CA* p.R88Q; Patient 2: *PIK3CA* p.R38H) and one with a second, C-terminal truncating *FGFR2* variant (Patient 3: *FGFR2* p.L776Rfs).

The sialoblastoma from Patient 5 did not have an *FGFR2* variant and instead showed a *CTNNB1* missense hotspot variant (*CTNNB1* p.I35T); the concurrent hepatoblastoma in this patient had a distinct *CTNNB1* hotspot variant (*CTNNB1* p.S45P).

Whole transcriptome sequencing was negative for recurrent fusion transcripts in one patient (Patient 1).

**Fig. 4:**
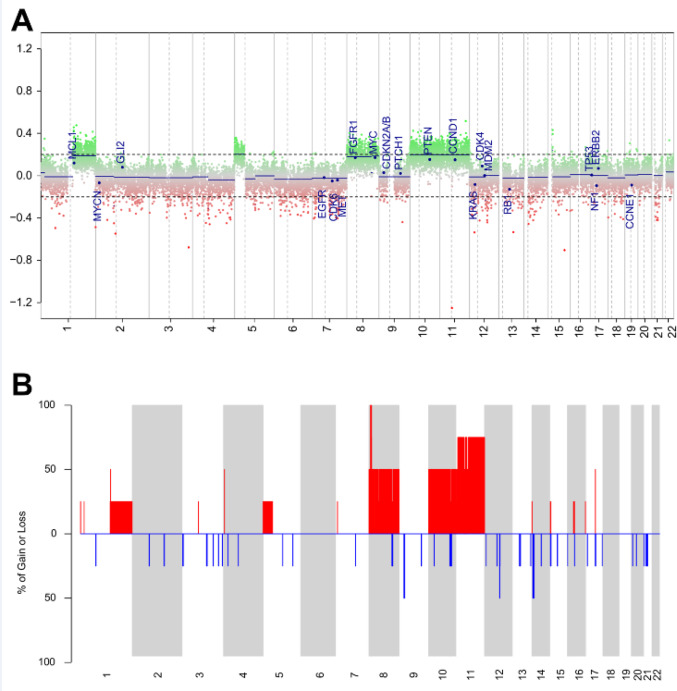
Chromosomal copy number changes in sialoblastoma. **A** Copy number plot for tumor from Patient 3 exhibits multiple whole-chromosome (+ 8, + 10, +11) and arm-level (+ 1q, +5p) gains. Green: chromosomal gain; red: chromosomal loss. **B** Cumulative copy number plot shows a recurrent pattern of copy number gains, including whole-chromosome gains of chromosomes 8, 10, and 11. Red: chromosomal gain; blue: chromosomal loss. Chromosome numbers are listed along the x-axis for both plots.

Copy number analysis by methylation array was performed on four of five cases (Patients 1, 3, 4, and 5) (Table 1; Fig. 4). Of the four cases, recurrent whole-chromosome gains of chromosomes 8 (2 cases), 10 (2 cases), and 11 (3 cases) were seen. No gene-specific copy number gains or losses (e.g. involving *MYC*,* CDKN2A/B*,* TERT*,* PTEN*,* MDM2*, or *RB1*) were identified. The tumor from Patient 3 additionally had apparently single-copy arm-level gains of 1q and 5p. The single *CTNNB1*-mutant tumor (Patient 5) showed no copy number alterations.

## Discussion

Our cohort of five sialoblastomas expands the molecular landscape of this rare pediatric salivary gland neoplasm (previous results summarized in Table 2). We identified recurrent *FGFR2* p.C382R variants in 80% (4/5) of our cases, with a subset harboring additional pathogenic alterations including *PIK3CA* hotspot mutations and a truncating *FGFR2* variant. Our *FGFR2-*mutant tumors displayed solid tumor architecture, primitive basaloid cytology, frequent necrosis, and an aggressive clinical course. These findings extend the recent observations by Jia et al., who reported *FGFR2* p.C382R mutations in five of eight sequenced sialoblastomas, with all *FGFR2*-mutant sialoblastomas demonstrating solid growth on histomorphology and exhibiting aggressive clinical behavior [4]. With the addition of the cases sequenced in this series, the *FGFR2* p.C382R variant is reported in 10 of 15 (67%) sialoblastomas with comprehensive sequencing to date (Tables 1 and 2).

**Table 1:** Clinical, histologic, and molecular characteristics of sialoblastoma

Patient	Location	Age at initial diagnosis / Sex	Additional clinical history	Size (cm)	Tumor necrosis	Mitoses per 10 HPF	Ki-67 proliferative index (%)	LVI	Capsular Invasion	*FGFR2* Alteration	Other Single Nucleotide Variants / Indel	Copy Number Changes
1	Lip commissure	2 months (detected congenitally) / Female	Multiple local recurrences	1.5	Present	16	60	Present	Absent	*FGFR2* p.C382R (VAF 97%)	*PIK3CA* p.R88Q (VAF 36%)	+ 8, + 10, +11
2	Parotid gland	3 months / Male	Cervical LAD	7.0	Present (focal, comedo-like)	25	40	Present	N/A	*FGFR2* p.C382R (VAF 54%)	*PIK3CA* p.R38H (VAF 34%)	N/A
3	Left buccal	Unknown / Female	Recurrent at time of biopsy (4 years of age)	N/A	Present (focal, comedo-like)	25	50	Absent	N/A	*FGFR2* p.C382R (VAF 60%) *FGFR2* p.L776Rfs (VAF 25%)	None	+ 1q, +5p, + 8, +10, + 11
4	Parotid gland	6 months / Female	N/A	N/A	Absent	8	40	Absent	Absent	*FGFR2* p.C382R (VAF 64%)	None	+ 11
5	Submandibular gland	2 months (detected congenitally) / Male	Concurrent hepatoblastoma (fetal type)	4.0	Absent	8	20	Absent	Absent	None	*CTNNB1* p.I35T (VAF 32%)	None

HPF: high-power fields; LAD: lymphadenopathy; LVI: lymphovascular invasion; N/A: not available; VAF: variant allele frequency

**Table 2:** Previously reported genetic alterations in sialoblastoma

Reference	Age	Location	Additional Clinical history	*FGFR2* Alteration	Other Sequencing Results
[31]	1 m	Submandibular	None	Unknown	47,XX, del(3)(q13),inv(9)(p11q12)c,+?r[5]/ 46,XX, inv(9)(p11q12)c[41]
[29]	6y	Parotid	None	Unknown	*MYB::NFIB* fusion negative *MAML2::CRTC1* fusion negative
[5]	N/A	N/A	Relapsed disease	*FGFR2* p.C382R	*TP53* p.R306*
[6]	6 m	Parotid	Synchronous hepatoblastoma	None reported	*PIK3CA* p.E545K 5q34-5q35.3 loss
[4]	5 m	Parotid	Locally recurrent, metastatic	*FGFR2* p.C382R	*PALB2* p.I265V *AR* p.F641C *MAP2K1* p.M308V
[4]	Birth	Cheek	Locally recurrent, metastatic	*FGFR2* p.C382R	None
[4]	Birth	Cheek	Locally recurrent, metastatic	*FGFR2* p.C382R	None
[4]	Birth	Cheek	Locally recurrent	*FGFR2* p.C382R	None
[4]	Birth	Parotid	Locally recurrent	*FGFR2* p.C382R	None
[4]	2.5y	Parotid	None	None	*HRAS* p.G13R
[4]	Birth	Submandibular	None	None	None
[4]	Birth	Parotid	None	None	None

*FGFR2* encodes fibroblast growth factor receptor 2, a receptor tyrosine kinase that activates downstream pathways involved in cell proliferation and differentiation. *FGFR2* p.C382R is a hotspot mutation that affects the transmembrane domain of the FGFR2 protein, causing constitutive activation of downstream signaling pathways [12]. This mutation is recurrent in several human malignancies, including cholangiocarcinoma and endometrial carcinoma [13, 14]. Activating *FGFR2* mutations, but not the p.C382R variant, are also reported in a subset of fusion-negative adenoid cystic carcinoma [15, 16]. Sensitivity to FGFR1-3 inhibitors such as pemigatinib has been reported in patients with *FGFR2* p.C382R mutant cholangiocarcinoma [17]. The prevalence of the *FGFR2* p.C382R mutation in sialoblastoma underscores the role of FGFR2 signaling in tumorigenesis and supports exploring targeted therapy in these rare tumors.

Secondary genomic alterations in addition to the driver *FGFR2* p.C382R mutations have rarely been documented in sialoblastoma [4, 5]. Within our cohort of four tumors with *FGFR2* p.C382R mutations, we identified concurrent *PIK3CA* mutations in two tumors and a second *FGFR2* mutation in another. Truncating *FGFR2* exon 18 mutations, such as the *FGFR2* frameshift variant seen in Tumor 3, are oncogenic and recurrent in cholangiocarcinoma and gastroesophageal and breast carcinoma and confer sensitivity to FGFR inhibition [18]. The *PIK3CA* p.R38H (Tumor 2) and p.R88Q (Tumor 1) variants both affect the adaptor-binding domain of the encoded protein; in vitro studies show gain of function through Akt signaling for both variants [19; 20], which are recurrent in carcinomas including squamous cell carcinoma and colonic adenocarcinoma [14, 21]. All three tumors with secondary genomic alterations showed aggressive behavior including local recurrence and lymph node involvement. Whether this behavior stems from synergistic effects of dual mutations or from the driver *FGFR2* mutation alone is unclear. While co-mutations of *FGFR2* with *PIK3CA* or truncating exon 18 *FGFR2* variants occur in other tumor types [22–24], these have not previously been reported in sialoblastoma.

Our study also provides the first reported copy number analysis in sialoblastoma, with three of four tumors showing recurrent whole-chromosome gains (+ 8, + 10, +11). Trisomies of chromosome 8 and/or 11 are recurrent genetic findings in multiple tumor types, including benign fibrous lesions [25], infantile fibrosarcoma [26], and Ewing sarcoma [27], but do not appear to affect prognosis in those tumors. One tumor (Patient 3) also had arm-level gains of 1q and 5p, but segmental gains were not recurrent findings in this small cohort. Overall, the significance of these copy number changes remains unclear, though the lack of focal gene-level gains or losses suggests that copy number changes are unlikely to be a genetic driver in sialoblastomas.

A smaller subset of sialoblastoma lacks *FGFR2* hotspot mutations, instead harboring alternative drivers [4]. The *CTNNB1*-mutant (p.I35T) tumor in our series was morphologically distinct, displaying more fibrous stroma, focal glandular differentiation, less primitive cytologic features, a lower mitotic rate, and a lower Ki-67 proliferative index compared to the *FGFR2*-mutant tumors. The morphology of this tumor aligns with the non-solid/basal cell adenoma histologic pattern purportedly associated with better prognosis [4]. Since the specific *CTNNB1* variant seen in this tumor, p.I35T, has been reported in up to 80% of basal cell adenomas [28], this case may actually represent a basal cell adenoma presenting in infancy. Notably, this patient’s concomitant hepatoblastoma harbored a distinct *CTNNB1* mutation (p.S45P). Germline sequencing was not possible for the patients in this cohort due to patient consent, and the tumor-only sequencing does not distinguish between germline and tumor-specific variants; however, the distinct variants in this patient’s two tumors implies the lack of a shared germline *CTNNB1* variant. While synchronous sialoblastoma and hepatoblastoma are well documented, comparative molecular analysis is limited to a single prior case [6] that similarly showed distinct molecular profiles in both tumors. Genetic factors contributing to the development of concurrent sialoblastoma and hepatoblastoma remain unclear but should be further evaluated.

This study provides analysis for single nucleotide variants and insertion-deletion variants in over 300 genes, as well as copy number variants, representing the largest comprehensively sequenced cohort of sialoblastoma to date. Although many salivary gland tumors are driven by gene rearrangements rather than single nucleotide variants, no recurrent fusions have been identified in sialoblastoma thus far [5, 29]. While we could not perform fusion testing in four of the five tumors in our retrospective cohort, the one tumor with whole transcriptome sequencing (Patient 1) was negative for recurrent fusion transcripts. We also compared the genomic profile of our sialoblastoma cohort with salivary gland anlage tumor, another congenital/infantile salivary gland neoplasm that is generally benign and morphologically distinct from sialoblastoma. In contrast to salivary gland anlage tumors, which frequently have RB1 loss [30], we did not detect *RB1* alterations by sequencing and copy number analysis.

Accumulation of molecular data across tumor types often requires re-evaluation of diagnostic criteria. The World Health Organization currently defines sialoblastomas by clinical and morphologic features alone: a primitive salivary gland malignancy with solid organoid nests presenting almost exclusively within the first few months of life [2]. All cases in our cohort met this definition. However, this definition allows for the possibility that some sialoblastomas may represent congenital variants of other salivary gland tumors, such as basal cell adenoma, basal cell adenocarcinoma, or solid-pattern adenoid cystic carcinoma. The genetic heterogeneity in this cohort and in the literature suggests that a subset of sialoblastomas, particularly the “non-solid” variants lacking *FGFR2* mutations, may represent congenital presentations of other salivary gland tumor types. Whether the *FGFR2*-mutant group of congenital salivary gland tumors constitutes a unique entity or represents congenital/infantile presentation of an established salivary gland malignancy (such as adenoid cystic carcinoma) may also need to be addressed. A comprehensive re-appraisal of sialoblastomas, while beyond the scope of this work, appears necessary.

## Conclusion

In summary, our cohort validates previous findings of the *FGFR2* p.C382R hotspot mutation as the predominant driver mutation in sialoblastoma. Sialoblastomas with this mutation show solid growth pattern, aggressive histology (necrosis, high Ki-67 index), and poor clinical behavior. The presence of multiple concurrent genomic alterations may affect prognosis in these tumors. A distinct subset harbors alternative drivers, corresponding to more favorable histology. Whether this subset represents true sialoblastoma or other salivary gland tumors presenting in infancy remains to be seen. Our findings suggest that molecular profiling has potential utility in refining the classification of congenital/infantile salivary gland tumors.

## Data Availability

Sequencing data is available from the corresponding author upon reasonable request.
